# Coloration enhancement in *Procambarus clarkii* crayfish through dietary supplementation of phycocyanin extracted from *Arthrospira platensis* BUUC1503

**DOI:** 10.14202/vetworld.2024.2899-2908

**Published:** 2024-12-19

**Authors:** Pisan Yodngam, Rachanimuk Hiransuchalert

**Affiliations:** 1 Marine Biotechnology Research Unit, Faculty of Marine Technology, Burapha University, Chanthaburi Campus, Chanthaburi 22170, Thailand; 2 Program of Creative Science and Innovation, Faculty of Science, Burapha University, Chonburi 20131, Thailand

**Keywords:** diet supplementation, phycocyanin, pigmentation enhancement, *Procambarus clarkii*

## Abstract

**Background and Aim:**

The effects of phycocyanin (PC) on pigmentation have been extensively studied in fish; however, its specific impact on crustaceans, particularly *Procambarus clarkii*, remains underexplored. This study aimed to evaluate the impact of PCs extracted from *Arthrospira platensis* BUUC1503 on the color enhancement of *P. clarkii* crayfish when added to food pellets.

**Materials and Methods:**

The following five dietary treatments were prepared: a control (without PC) and four experimental diets with PC supplementation at concentrations of 10, 50, 100, and 500 mg/kg. The stability of the pellets in water was ensured for 180 min. *P. clarkii* crayfish were fed these diets at 5% of their body weight daily for 10 weeks. Body weight and total length were measured. Color changes in *P. clarkii* were also assessed. After the experiments, we treated the samples according to Animal Welfare Technical Information No.16.8 Correct euthanasia of decapods.

**Results:**

Although PC supplementation did not significantly affect the yellowness (b* value) of *P. clarkii*, it significantly enhanced the redness (a* value) and lightness (L* value) pigmentation compared with the control. At week 10, crayfish fed a diet containing 100 mg/kg PC exhibited the highest L* value (36.51 ± 1.59) (p < 0.001). Diet with 500 mg/kg PCs had the highest a* value (26.96 ± 0.64) (p < 0.001). Crayfish fed 50, 100, or 500 mg/kg PC had the highest survival rate (100%). PCs appear to positively affect weight gain in *P. clarkii* crayfish during weeks 8, 9, and 10 (p = 0.013, 0.003, and 0.034, respectively). However, statistical analysis revealed no significant differences in length among the dietary treatments.

**Conclusion:**

PCs effectively weight-gain and enhance red pigment deposition in the epidermal layer of *P. clarkii*. Further investigation is warranted to elucidate the underlying mechanisms.

## Introduction

*Procambarus clarkii* (Girarad, 1852) freshwater crayfish are native to the southcentral United States and northeastern Mexico [1]. They were introduced to Thailand in 1987 for both ornamental use and as a food option, with potential for large-scale farming [2]. Aquatic animal importers from abroad present a positive opportunity, as these species often thrive and reproduce successfully in Thailand. Farmers can capitalize on this opportunity by integrating these crayfish into their farming practices, increasing their income as new protein sources, and fostering the development of aquaculture industries for export and distribution. However, introducing foreign aquatic species may pose challenges if they negatively impact native aquatic flora and fauna or local resources.

*P. clarkii* crayfish is valued both as an ornamental species and for its potential use in aquaculture. In addition, studies have demonstrated that *P. clarkii* crayfish exhibit sensitivity to polluted water conditions, with dissolved oxygen levels at 2.55 ± 0.25 mg/L and biological oxygen demand at 8.45 ± 0.04 mg/L. These crayfish struggle to survive in such environments, restricting their ability to proliferate into other water bodies throughout Thailand. Biological control measures have indicated that *Anabas testudineus*, commonly known as the climbing perch, emerges as the most efficient predator, potentially aiding in resisting the invasion of exotic crayfish in natural habitats [2].

China is the world’s largest producer and exporter of crayfish. In 2015, China produced 723,207 tons (91.85%) of crayfish belonging to the genus *Procambarus* [3]. The global farmed crayfish production segment was valued at US$ 4,230.44 million in 2017 and has continued to grow. Due to the increased demand for fish protein from aquaculture consumers worldwide, farmers are focusing more on farmed varieties to sustain the market. Cost-effective aquaculture techniques are accelerating the expansion of the crayfish market. Among these, crayfish farming in cultivated forage ponds or within double-crop rotation systems, particularly in rice fields, is the most prevalent method. Successful crayfish farming requires access to high-quality water and land with clay contents exceeding 20%. In addition, efficient farming requires appropriate equipment and bait for harvesting, labor, electricity, and fuel. Fortunately, the necessary equipment and supplies are readily available from local sources, mitigating the need for substantial investments. These factors are expected to drive market growth throughout the forecast period [4]. The success of decapod farming relies heavily on various available diets. Present commercial practices involve providing juvenile decapods with a range of food options, including natural, live, and formulated feeds, as is done with pacific white shrimp (*Litopenaeus vannamei*), portunid crabs (*Scylla* spp. and *Portunus* spp.), and giant river prawns (*Macrobrachium rosenbergii*) [5]. This feeding regimen, known as exogenous feeding, refers to the provision of feed to caged decapods by farm practitioners.

Regarding ornamental animals, skin coloration plays a pivotal role in determining esthetic and market value. Crustacean pigmentation is predominantly due to the absorption of carotenoids from the diet. Synthetic and natural carotenoid pigments are used to enhance coloration, but synthetic options are often costly. To address this need, natural carotenoids are incorporated into aquaculture feed [6]. The use of protein pigments derived from algae to enhance skin color in animals, particularly aquaculture and ornamental species, has garnered significant attention due to its natural origins and effectiveness [7, 8]. Phycobiliproteins, particularly phycocyanin (PC) from *Spirulina* spp. and phycoerythrin from red algae are among the most studied pigments for these purposes [9].

PC, a non-carotenoid pigment, is responsible for imparting blue coloration in fish. This pigment is synthesized by blue-green algae, which are commonly found in spirulina preparations [10]. In ornamental fish, such as koi (*Cyprinus carpio* var. koi), diets containing PC exhibit significantly higher growth rates than those containing carotenoids. Incorporating PC into feed has been linked to improved coloration, a desirable trait in ornamental species, such as betta fish and crayfish [11, 12]. This pigment not only enhances the esthetic appeal but also has antioxidant properties, contributing to the overall health of animals [13]. In addition to their esthetic benefits, protein pigments from algae are preferred because of their natural and sustainable origins, offering an eco-friendly alternative to synthetic dyes. This is particularly important in industries where consumer demand is shifting toward natural products that improve animal well-being and environmental sustainability [14].

PC is a natural pigment found in cyanobacteria, such as *Spirulina* spp., and has gained significant attention for its aquaculture applications. As a phycobiliprotein, PC imparts a distinctive blue-green color and exhibits strong antioxidant and anti-inflammatory properties, making it a valuable additive in animal feed [15]. In ornamental aquaculture, coloration is a critical factor influencing the market value of species, particularly in crustaceans like *P. clarkii* (red swamp crayfish), which are prized for their vibrant hues.

The mechanism by which these pigments enhance coloration is largely attributed to their ability to accumulate in the skin and scale tissues of animals, where they interact with other pigments and light to produce vivid colors [16]. In addition, these pigments can act as precursors for other pigments, such as carotenoids, further contributing to enhanced coloration [6]. Pigmentation is correlated with the dose of dietary-supplemented carotenoids and PCs, with carotenoids having a greater influence on fish pigmentation than PC [11]. The effects of PC on crayfish pigmentation have not been investigated. In contrast to previous studies [11, 12], our research specifically addressed the impact of PC on the pigmentation of *P. clarkii* crayfish, a crustacean species for which the pigmentation effects of PC have not been documented. While Li *et al*. [6] have demonstrated that dietary pigments, including carotenoids and PC, enhance coloration primarily in fish species, our study extends this understanding to a new taxonomic group within crustaceans.

A key novelty of our study was the evaluation of the accumulation and specific color-enhancing effects of PC on the lightness (L*), redness (a*), and yellowness (b*) of crayfish, demonstrating the dose-dependent pigmentation effects previously unreported in crayfish.

## Materials and Methods

### Ethical approval

The researchers who handled and treated the animals in this study were certified by the Institute of Animals for Scientific Purposes Development (IAD), Thailand, under Animal Use Certificate No. U1–03631-2559, confirming that they completed the required training for ethical animal handling and care at the national level.

### Study period and location

This study was conducted from January to June 2020 at Marine Biotechnology Research Unit, Faculty of Marine Technology, Burapha University, Chanthaburi campus, Thailand.

### Experimental animals

Seven- to ten-day-old *P. clarkii* crayfish, with an average size of 1 cm (n = 20), were cultured separately in glass tanks containing 3 L of fresh water (30 × 15 × 20 cm; water height of 0.5 m) for 14 days to allow acclimation to the experimental environment. All *P. clarkii* crayfish were fed an equal amount of diet during this experimental period (5% of body weight).

### Green extraction of PC from *A. platensis* BUUC1503

PC was extracted from *A. platensis* BUUC1503 (kindly provided by Marine Biotechnology Research Unit, Faculty of Marine Technology, Burapha University, Chanthaburi) using the freeze-thawing method [17, 18]. With a minor modification based on a previous study [19]; briefly, 1 g of *A. platensis* BUUC1503 was mixed with 20 mL of 0.1 M Na-phosphate buffer (pH 7.0). The solution was frozen at −20°C for 12 h and then thawed at 25°C for 12 h. The freeze-thawing cycle was repeated twice, and the solution was filtered through 54 sheets of circular filter paper (Maxmo, Thailand; 2 cm thickness and 3 cm in diameter) packed in a 50-mL syringe. The PC solution was passed through a syringe to allow the PC to flow through the filter paper. The PC solution was further purified using medicinal charcoal tablets (DeltaCarbon, South Africa) at a concentration of 2000 mg/20 mL of PC solution. The solution was then stirred for 10 min and then filtered using a syringe containing filter paper, as described above. This filtering process was repeated using a cellulose filter paper (Whatman^®^ Filter Paper No.1; Whatman™, Cytiva, USA). The absorbance was measured at wavelengths of 280, 562, 620, and 652 nm to determine the C-PC concentration using the following equation: C-PC (mg⁄mL) = [A620 nm – 0.474 (A652 nm)]/(5.34). C-PC purity was determined using the following equation: Purity=A 620⁄A 280.

### Preparation of a PC-supplemented diet for *P. clarkii*

Four treatment groups of diets were prepared using commercial crayfish feed (2.0 mm pellet size, 31.0% crude protein minimum, 0.7% phosphorus, 19,000 IU/kg vitamin A, 2,900 IU/kg vitamin D3, 600 IU/kg vitamin E, 420 mg/kg ascorbic acid, and 0.7% calcium; Crab Cuisine^®^, Hikari, Japan) and different concentrations of PC (10 mg/kg, 50 mg/kg, 100 mg/kg, and 500 mg/kg). Additionally, a control group was included, which received the same commercial crayfish feed without any PC supplementation. The selected PC concentrations were based on previous studies that examined the effects of dietary pigments on aquaculture species, particularly ornamental fish and crustaceans [6, 11, 12]. The experimental feeds were spread on an aluminum tray, and a solution of each experimental formula was mixed by spraying over the pelleted diet. All experimental feeds were air-dried overnight in a dark ventilated room. After drying, the experimental feeds were stored at −20°C until use.

### Physical change test of diet

The physical changes of *P. clarkii* crayfish diets were evaluated after being soaked in water for 90 min: (1) standard crayfish diet, (2) crayfish diet coated with squid liver oil (Vemedim Corporation, Vietnam) [100:1, pellets: oil (W/V)], (3) crayfish diet mixed with PC, and (4) crayfish diet mixed with PC and coated with squid liver oil (Vemedim Corporation) [100:1, pellets: oil (W/V)].

### Feed proximate composition

The proximate composition of the feed was determined according to the standard methods of the Association of Official Agricultural Chemists [20]. The moisture content of the feed was determined by weighing the sample, oven-drying the sample to a constant weight at 103 ± 2°C and calculated the percentage of water in the sample. The ash content was determined by weighing the samples, searing them in a muffle furnace at 550°C, and calculated the percentage of ash in each sample. Crude protein content was determined using the Kjeldahl nitrogen method (K-360; Buchi Labortechnik Ag., Flawil, Switzerland). Crude lipid content was measured using a Soxtherm device (B-811; Buchi Labortechnik Ag.). Vitamin C and E contents were measured by high-performance liquid chromatography (E2695; Waters Co., Ltd., Milford, MA, USA). Amino acid content was measured using an amino acid analyzer (L-8080; Hitachi Ltd., Tokyo, Japan).

### Examination of color changes in *P. clarkii* crayfish

Color changes in *P. clarkii* crayfish (n = 4 for each treatment) were assessed by measuring the color on both sides of the shrimp body weekly using a colorimeter (CR-400/CR-410) (Konica Minolta, Inc., Japan). The L* (lightness), a* (redness), and b* (yellowness) values were recorded. The growth of crayfish was also monitored by measuring body weight and total length weekly (70 days).

### Statistical analysis

The statistical analyses of body weight, total length, and color of *P. clarkii* crayfish were performed using Microsoft Excel (Office 365) version 16.78.3 (Microsoft Corp., Washington, USA). p < 0.05 was considered statistically significant.

## Results

### PC content of *A. platensis*

PC was extracted from *A. platensis* via purification steps that progressively increased its purity. The concentration of PC was 1.09 ± 0.02 mg/mL, and the final purity was 1.25.

### Physical changes in PC-supplemented diets

**Figure-1:**
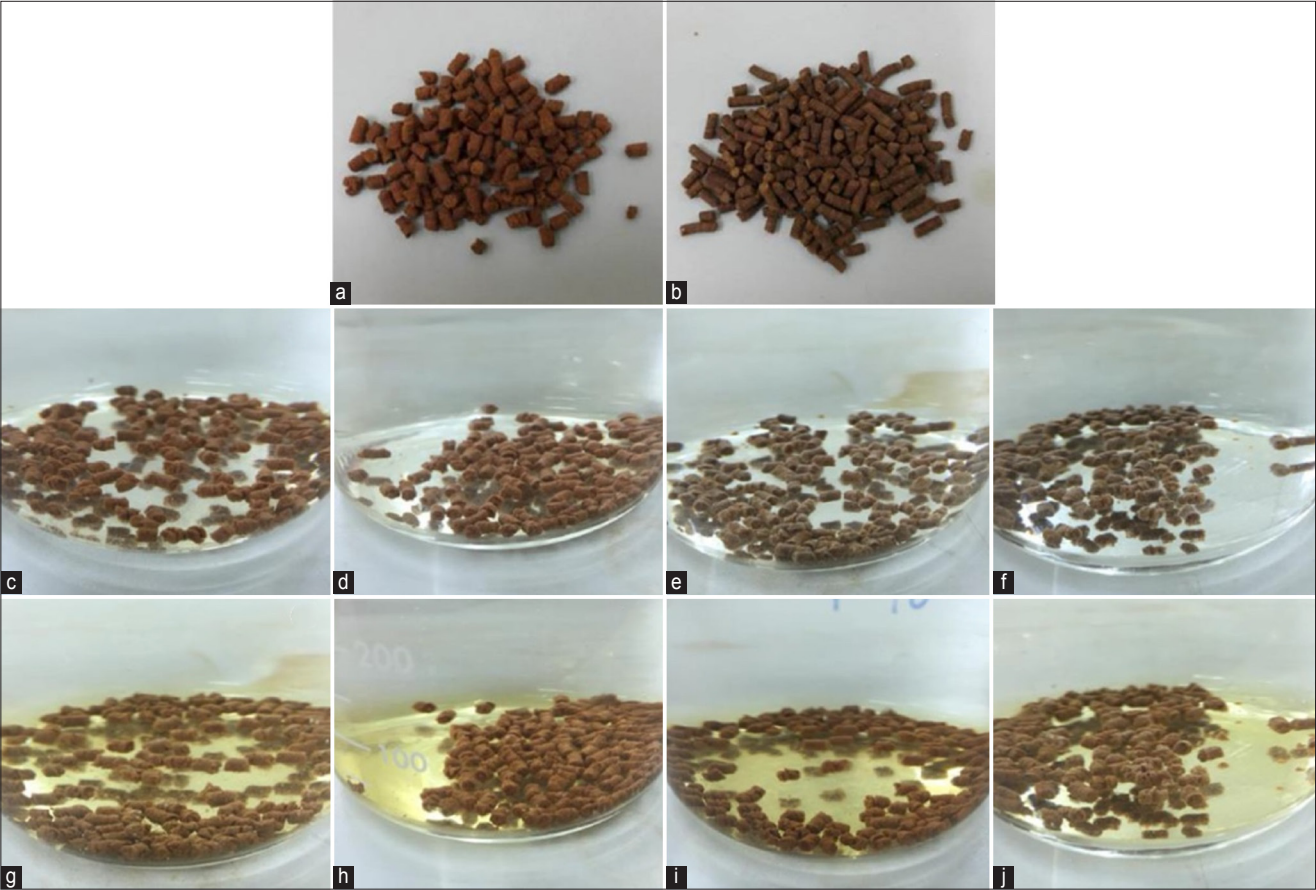
Characteristics of the pellets (a) before mixing with phycocyanin and (b) after mixing with phycocyanin. Physical changes in crayfish feed at (c–f) 0 min and (g–j) after 90 min of soaking in water. Set 1 (c and g), a standard crayfish diet; Set 2 (d and h), a diet with squid oil coating; Set 3 (e and i), a diet supplemented with phycocyanin; and Set 4 (f and j), a diet with both phycocyanin supplementation and squid oil coating.

The physical change test results during the soaking period are summarized in Figure-1. The standard crayfish diet (Set 1) began to swell after 60 min, showing a different water persistence than in the other sets. The squid oil-coated diet (Set 2), PC-supplemented diet (Set 3), and the squid oil-coated PC-supplemented diet (Set 4) began to swell after 90 min but maintained a better structure over time, with only slight swelling and dissolution. These results indicate that the addition of squid ink oil and PC affects the physical stability of crayfish feed in water, with the combination of PC and squid oil coating (Set 4) showing the best structural integrity and least dissolution over time.

### Nutritional analysis of the diet

The nutritional composition of each diet was analyzed, and the results are presented in Table-1. The protein content in the diets ranged from 45.75% to 46.27%, whereas the fat content ranged from 8.93% to 10.61%. These results indicated that the addition of PC to the diet slightly influenced the nutritional composition, particularly in increasing fat content as the concentration of PC increased. In addition, the results presented in Table-1 indicate that the diets exhibited variable moisture levels, with the highest moisture content observed in the control and 500 mg/kg PC groups. The ash content was consistent across all diets.

**Table-1:** Nutritional value (Mean ± standard deviation) of phycocyanin-supplemented diets.

Experimental group	Moisture (%)	Ash (%)	Fat (%)	Protein (%)	Fiber (%)
0 mg/kg (Control)	8.21 ± 0.06	13.47 ± 0.22	8.93 ± 0.81	45.75 ± 0.06	0.93 ± 0.01
10 mg/kg	6.90 ± 0.53	13.67 ± 0.29	9.72 ± 0.85	45.77 ± 0.06	0.94 ± 0.01
50 mg/kg	4.62 ± 0.03	13.57 ± 0.47	10.62 ± 0.29	46.13 ± 0.03	0.95 ± 0.01
100 mg/kg	4.34 ± 0.24	13.85 ± 0.31	9.68 ± 1.34	46.17 ± 0.02	0.96 ± 0.00
500 mg/kg	9.02 ± 0.43	13.33 ± 0.29	10.61 ± 0.76	46.27 ± 0.10	0.99 ± 0.01

### Weight and length of *P. clarkii* crayfish

The crayfish were supplemented for 10 weeks, during which they were weighed weekly to monitor growth changes. PC appears to positively affect weight gain in *P. clarkii* crayfish during weeks 8–10 (p = 0.013, 0.003, and 0.034, respectively) (Table-2). In these weeks, crayfish fed PC-supplemented diets, particularly at higher doses, showed a statistically significant increase in weight compared with the control group. This suggests that PC may have a growth-promoting effect that becomes more pronounced over time, enhancing weight gain in the later stages of the study.

**Table-2:** Weekly weight gain (Mean ± standard deviation) of *P. clarkii* crayfish fed phycocyanin-supplemented diets.

Week	Weight (g) of *P. clarkii* crayfish					p-value

	Control (0 mg/kg)	10 mg/kg	50 mg/kg	100 mg/kg	500 mg/kg	
0	2.20 ± 0.05^a^	2.20 ± 0.04^a^	2.18 ± 0.06^a^	2.19 ± 0.06^a^	2.13 ± 0.08^a^	0.436
1	2.23 ± 0.04^a^	2.25 ± 0.03^a^	2.24 ± 0.04^a^	2.19 ± 0.08^a^	2.21 ± 0.05^a^	0.435
2	2.38 ± 0.06^a^	2.39 ± 0.06^a^	2.43 ± 0.04^a^	2.37 ± 0.07^a^	2.44 ± 0.06^a^	0.351
3	3.12 ± 0.03^a^	3.16 ± 0.03^ab^	3.19 ± 0.03^bc^	3.24 ± 0.03^c^	3.21 ± 0.04^bc^	0.001
4	4.24 ± 0.07^a^	4.28 ± 0.06^a^	4.37 ± 0.09^a^	4.36 ± 0.08^a^	4.26 ± 0.10^a^	0.131
5	4.95 ± 0.16^a^	4.99 ± 0.12^a^	5.03 ± 0.19^a^	5.02 ± 0.25^a^	5.11 ± 0.16^a^	0.781
6	5.43 ± 0.17^a^	5.45 ± 0.23^a^	5.54 ± 0.14^a^	5.63 ± 0.09^a^	5.53 ± 0.09^a^	0.375
7	6.10 ± 0.06^a^	6.16 ± 0.05^a^	6.16 ± 0.08^a^	6.20 ± 0.05^a^	6.13 ± 0.09^a^	0.273
8	6.47 ± 0.11^a^	6.57 ± 0.09^abc^	6.64 ± 0.05^c^	6.68 ± 0.06^c^	6.57 ± 0.05^abc^	0.013
9	7.05 ± 0.08^a^	7.13 ± 0.05^ab^	7.19 ± 0.06^bc^	7.25 ± 0.04^b^	7.22 ± 0.06^b^	0.003
10	7.47 ± 0.10^a^	7.58 ± 0.08^ab^	7.62 ± 0.09^ab^	7.73 ± 0.09^b^	7.66 ± 0.14^ab^	0.034

The superscript letters assigned to each row indicate statistically significant differences at p < 0.05. *P. clarkii*=*Procambarus clarkii*

The results showed an increase in *P. clarkii* crayfish length across all diet groups, but statistical analysis revealed no significant differences in length between the different dietary treatments (Table-3). These results suggested that although crayfish grew over 10 weeks, the addition of PC at the tested concentrations did not significantly impact their growth in length.

**Table-3:** Weekly length gain (Mean ± standard deviation) in *P. clarkii* crayfish fed phycocyanin-supplemented diets.

Week	Length (cm) of *P. clarkii* crayfish					p-value

	Control (0 mg/kg)	10 mg/kg	50 mg/kg	100 mg/kg	500 mg/kg	
0	1.53 ± 0.10^a^	1.55 ± 0.10^a^	1.53 ± 0.10^a^	1.48 ± 0.05^a^	1.53 ± 0.10^a^	0.823
1	1.75 ± 0.06^a^	1.75 ± 0.13^a^	1.78 ± 0.05^a^	1.75 ± 0.06^a^	1.75 ± 0.13^a^	0.993
2	2.15 ± 0.06^a^	2.15 ± 0.10^a^	2.18 ± 0.10^a^	2.18 ± 0.10^a^	2.28 ± 0.17^a^	0.497
3	2.48 ± 0.05^a^	2.53 ± 0.10^a^	2.55 ± 0.06^a^	2.48 ± 0.10^a^	2.60 ± 0.14^a^	0.324
4	2.85 ± 0.06^a^	2.88 ± 0.05^a^	2.88 ± 0.05^a^	2.93 ± 0.05^a^	3.03 ± 0.22^a^	0.220
5	3.58 ± 0.13^a^	3.63 ± 0.05^a^	3.63 ± 0.10^a^	3.63 ± 0.10^a^	3.63 ± 0.13^a^	0.940
6	4.15 ± 0.06^a^	4.13 ± 0.05^a^	4.20 ± 0.08^a^	4.23 ± 0.10^a^	4.20 ± 0.08^a^	0.355
7	4.33 ± 0.05^a^	4.35 ± 0.06^a^	4.40 ± 0.08^a^	4.45 ± 0.06^a^	4.45 ± 0.10^a^	0.085
8	4.65 ± 0.06^a^	4.70 ± 0.08^a^	4.73 ± 0.10^a^	4.68 ± 0.10^a^	4.73 ± 0.10^a^	0.691
9	5.03 ± 0.15^a^	5.08 ± 0.13^a^	5.15 ± 0.06^a^	5.15 ± 0.06^a^	5.18 ± 0.05^a^	0.212
10	5.45 ± 0.06^a^	5.43 ± 0.05^a^	5.45 ± 0.06^a^	5.45 ± 0.06^a^	5.50 ± 0.08^a^	0.554

The superscript letters assigned to each row indicate statistically significant differences at p < 0.05. *P. clarkii*=*Procambarus clarkii*

**Figure-2:**
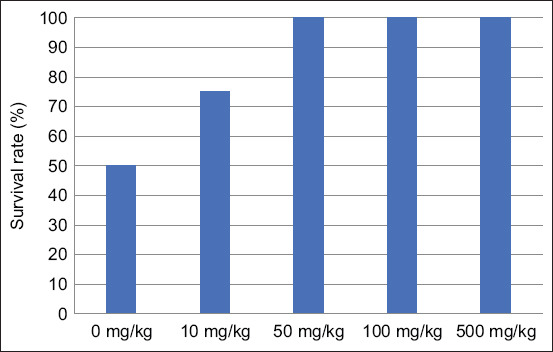
Survival rate (%) of *Procambarus clarkii* crayfish after feeding diets containing different concentrations of phycocyanins over a specified period. The five groups in the study included a control group (no phycocyanin) and four experimental groups with phycocyanin concentrations of 10, 50, 100, and 500 mg/kg.

Figure-2 shows that crayfish fed the control diet (0 mg/kg PC) had the lowest survival rate (50%), whereas the survival rate increased when the concentration of PC in the diet increased. The survival rate of crayfish fed 10 mg/kg PC was 75%; interestingly, crayfish fed 50, 100, and 500 mg/kg PC had the highest survival rates (100%), indicating a positive correlation between the concentration of PC in the diet and the survival rate of *P. clarkia* crayfish. The survival rate of crayfish approached 100% as the concentration of PC increased, demonstrating the beneficial effects of PC on crayfish health. Specifically, the experimental groups receiving PC concentrations of 50, 100, and 500 mg/kg all exhibited 100% survival rates, suggesting that PC supplementation supports improved resilience and health outcomes in *P. clarkii*.

Lightness (L* value), redness (a* value), and yellowness (b* values) of *P. clarkii* crayfish fed different diets.

**Figure-3:**
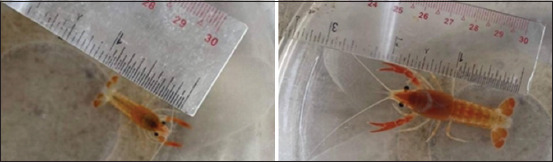
*Procambarus clarkii* crayfish at (a) the beginning and (b) end of the study.

The present study examined the effects of different PC-supplemented diets on the color attributes of crayfish, specifically focusing on lightness (L* value), redness (a* value), and yellowness (b* value) (Figure-3).

The L* lightness values of *P. clarkii* crayfish fed the 100 and 500 mg/kg PC diets were significantly higher during the 6 weeks than those fed the control diet, 10 mg/kg PC, and 50 mg/kg PC diets (p < 0.001) (Table-4). From weeks 7 to 10, *P. clarkii* crayfish fed the 100 and 500 mg/kg PC diets exhibited the highest luminance intensity, with significantly greater values than those of crayfish fed the 10, 50, and control diets (p < 0.001). *P. clarkii* crayfish fed the 500 mg/kg PC diet consistently showed significantly higher a* redness values from weeks 2 to 10 compared to all other diets (p < 0.001) (Table-5). Additionally, from week 5 to 10, *P. clarkii* crayfish fed the 100 mg/kg PC diet also showed significantly greater redness than those fed the control, 10 mg/kg PC, and 50 mg/kg PC diets, but the redness was still less than that of *P. clarkii* crayfish fed the 500 mg/kg PC diet (p < 0.001). There was no significant difference in b* yellowness values between the different diet groups during the 10 weeks of the experiment (p = 0.050–0.979) (Table-6).

**Table-4:** Comparison of luminance values (L* lightness values) (Mean ± standard deviation) of *P. clarkii* crayfish fed five phycocyanin-supplemented diets.

Week	L* lightness values					p-value

	Control (0 mg/kg)	10 mg/kg	50 mg/kg	100 mg/kg	500 mg/kg	
0	14.65 ± 1.21^a^	15.22 ± 0.95^a^	14.32 ± 0.66^a^	16.89 ± 1.69^a^	19.17 ± 0.41^b^	<0.001
1	15.99 ± 0.44^ab^	16.29 ± 1.44^ab^	15.21 ± 0.55^bc^	22.72 ± 4.74^a^d	20.18 ± 1.15^ab^	0.001
2	17.82 ± 1.13^a^	17.86 ± 1.34^a^	16.18 ± 0.89^a^	27.33 ± 2.29^b^	23.46 ± 3.40^b^	<0.001
3	19.22 ± 1.46^a^	18.77 ± 1.50^a^	17.01 ± 1.05^a^	26.32 ± 5.71^b^	26.93 ± 1.16^b^	<0.001
4	20.15 ± 1.12^a^	20.99 ± 2.57^a^	18.10 ± 0.99^a^	37.68 ± 1.83^b^	19.52 ± 0.62^a^	<0.001
5	21.16 ± 0.83^a^	21.31 ± 2.49^a^	18.35 ± 0.76^a^	30.96 ± 1.92^b^	18.35 ± 0.76^a^	<0.001
6	22.19 ± 1.17^ab^	22.16 ± 2.45^ab^	18.75 ± 0.77^ab^	26.95 ± 1.15^c^	24.51 ± 3.10^ab^	<0.001
7	19.40 ± 0.92^a^	22.62 ± 2.20^a^	19.10 ± 0.59^a^	28.35 ± 0.76^b^	29.18 ± 3.93^b^	<0.001
8	22.89 ± 0.59^a^	20.92 ± 2.68^a^	19.30 ± 0.64^a^	27.02 ± 4.54^ab^	31.44 ± 6.14^bc^	0.001
9	19.33 ± 0.74^ab^	19.23 ± 1.10^ab^	19.02 ± 0.66^ab^	32.68 ± 6.14^c^	36.43 ± 1.78^c^	<0.001
10	19.95 ± 0.30^a^	19.73 ± 1.36^a^	19.28 ± 0.79^a^	36.51 ± 1.59^b^	34.37 ± 4.11^b^	<0.001

The superscript letters assigned to each data set indicate statistically significant differences at p < 0.05. *P. clarkii*=*Procambarus clarkii*

**Table-5:** Comparison of a* redness values (Mean ± standard deviation) of *P. clarkii* crayfish fed five phycocyanin-supplemented diets.

Week	a* redness values					p-value

	Control (0 mg/kg)	10 mg/kg	50 mg/kg	100 mg/kg	500 mg/kg	
0	3.56 ± 0.67^a^	4.06 ± 0.56^a^	3.29 ± 0.79^a^	3.25 ± 0.73^a^	4.49 ± 0.30^a^	0.060
1	4.66 ± 1.13^ab^	6.46 ± 0.34^bc^	4.44 ± 1.76^ab^	4.05 ± 0.95^ad^	6.33 ± 0.58^ab^	0.015
2	6.31 ± 0.86^a^	7.17 ± 0.38^a^	5.38 ± 1.68^a^	5.50 ± 1.89^a^	14.67 ± 0.22^b^	<0.001
3	7.39 ± 1.07^a^	7.31 ± 0.31^a^	6.04 ± 1.65^a^	6.60 ± 2.33^a^	16.68 ± 0.32^b^	<0.001
4	8.20 ± 0.48^a^	9.50 ± 0.26^a^	7.49 ± 2.32^a^	8.60 ± 3.03^a^	21.9 ± 0.56^b^	<0.001
5	9.10 ± 0.50^a^	9.63 ± 0.29^ab^	8.74 ± 2.46^a^	12.34 ± 1.70^b^	22.04 ± 0.90^c^	<0.001
6	10.10 ± 0.72^a^	10.90 ± 1.14^a^	9.39 ± 2.21^a^	14.60 ± 1.79^b^	22.87 ± 0.67^c^	<0.001
7	11.40 ± 1.47^a^	11.78 ± 0.64^a^	10.80 ± 2.76^a^	17.01 ± 2.88^b^	23.34 ± 0.51^c^	<0.001
8	13.05 ± 2.42^a^	13.04 ± 1.57^a^	12.17 ± 3.65^a^	19.51 ± 2.79^b^	25.10 ± 0.92^c^	<0.001
9	13.64 ± 2.26^a^	14.64 ± 1.66^a^	15.76 ± 2.25^ac^	21.58 ± 3.05^bc^	26.40 ± 0.68^d^	<0.001
10	15.25 ± 2.76^a^	16.81 ± 1.51^a^	18.23 ± 3.32^a^	25.35 ± 0.00^b^	26.96 ± 0.64^b^	<0.001

The superscript letters assigned to each data set indicate statistically significant differences at p < 0.05. *P. clarkii*=*Procambarus clarkii*

**Table-6:** Comparison of yellow intensity (b* yellowness values) (Mean ± Standard deviation) of *P. clarkii* crayfish fed five phycocyanin-supplemented diets.

Week	b* yellowness values					p-value

	Control (0/mg/kg)	10 mg/kg	50 mg/kg	100 mg/kg	500 mg/kg	
0	22.11 ± 1.13^a^	21.42 ± 0.00^a^	21.09 ± 0.58^a^	21.94 ± 0.37^a^	20.55 ± 0.94^a^	0.050
1	24.51 ± 3.10^a^	24.69 ± 0.35^a^	24.36 ± 0.71^a^	25.18 ± 1.18^a^	24.35 ± 0.17^a^	0.935
2	26.01 ± 3.30^a^	26.29 ± 0.40^a^	26.59 ± 0.57^a^	27.72 ± 0.76^a^	26.09 ± 0.65^a^	0.552
3	27.15 ± 3.16^a^	27.91 ± 0.36^a^	28.03 ± 0.25^a^	27.95 ± 0.75^a^	27.00 ± 1.08^a^	0.803
4	29.18 ± 3.93^a^	29.09 ± 1.12^a^	29.29 ± 1.16^a^	29.54 ± 1.47^a^	29.03 ± 1.11^a^	0.100
5	29.59 ± 3.66^a^	29.59 ± 1.56^a^	29.56± 1.47^a^	30.19 ± 2.05^a^	29.27 ± 1.36^a^	0.979
6	29.87 ± 3.46^a^	30.41 ± 1.23^a^	30.56 ± 1.59^a^	30.67 ± 1.72^a^	29.92 ± 0.96^a^	0.966
7	30.65 ± 3.77^a^	31.06 ± 1.55^a^	31.20 ± 1.45^a^	31.20 ± 2.29^a^	30.37 ± 1.15^a^	0.977
8	30.79 ± 4.24^a^	31.45 ± 1.56^a^	31.90 ± 1.91^a^	32.25 ± 2.00^a^	30.94 ± 1.08^a^	0.898
9	31.01 ± 3.77^a^	32.07 ± 1.25^a^	32.61 ± 1.80^a^	33.22 ± 1.96^a^	31.82 ± 1.67^a^	0.708
10	31.43 ± 4.04^a^	32.88 ± 0.96^a^	33.47 ± 1.366^a^	33.90 ± 1.49^a^	31.13 ± 1.52^a^	0.323

The superscript letters assigned to each data set indicate statistically significant differences at p < 0.05. *P. clarkii*=*Procambarus clarkii*

Comparison of the differences in L* lightness, a* redness, and b* yellowness values of all experimental *P. clarkii* crayfish at weeks 1 and 10 demonstrated that these values increased significantly throughout the experiment (p < 0.05) (Table-7). The L* lightness of *P. clarkii* crayfish fed the PC-supplemented diets was higher than that of *P. clarkii* crayfish fed the control diet at week 10. *P. clarkii* crayfish fed the 100 mg/kg PC diet exhibited the highest luminance, indicating a significant enhancement in brightness. For a* redness values, *P. clarkii* crayfish fed the 500 mg/kg PC diet showed the highest red intensity by the end of week 10, suggesting that a higher PC concentration enhances red pigmentation. PC supplementation did not significantly affect the yellowness (b* value) of *P. clarkii* crayfish.

**Table-7:** Comparison of differences in color intensity (Mean ± standard deviation) among *P. clarkii* crayfish fed five phycocyanin-supplemented diets.

Intensity	Week	Control (0 mg/kg)	10 mg/kg	50 mg/kg	100 mg/kg	500 mg/kg	p-value
L* lightness values	0	14.65 ± 1.21^a^	15.22± 0.95^a^	14.32±0.66^a^	16.89 ± 1.69^a^	19.17 ± 0.41^b^	<0.001
	1	15.99 ± 0.44^ab^	16.29±1.44^ab^	15.21±0.55^bc^	22.72 ± 4.74^ad^	20.18 ± 1.15^ab^	0.001
	10	19.95 ± 0.30^a^	19.73± 1.36^a^	19.28± 0.79^a^	36.51 ± 1.59^b^	34.37 ± 4.11^b^	<0.001
a* redness values	0	3.56 ± 0.67^a^	4.06 ± 0.56^a^	3.29 ± 0.79^a^	3.25 ± 0.73^a^	4.49 ± 0.30^a^	0.060
	1	4.66 ± 1.13^ab^	6.46 ± 0.34^bc^	4.44 ± 1.76^ab^	4.05 ± 0.95^ad^	6.33 ± 0.58^ab^	0.015
	10	15.25 ± 2.76^a^	16.81 ± 1.51^a^	18.23 ± 3.32^a^	25.35 ± 0.00^b^	26.96 ± 0.64^b^	<0.001
b* yellowness values	0	22.11 ± 1.13^a^	21.42 ± 0.00^a^	21.09 ± 0.58^a^	21.94 ± 0.37^a^	20.55 ± 0.94^a^	0.050
	1	24.51 ± 3.10^a^	24.69 ± 0.35^a^	24.36 ± 0.71^a^	25.18 ± 1.18^a^	24.35 ± 0.17^a^	0.935
	10	31.43 ± 4.04^a^	32.88 ± 0.96^c^	33.47 ± 1.366^d^	33.90 ± 1.49^bc^	31.13 ± 1.52^b^	0.323

The superscript letters assigned to each data set indicate statistically significant differences at p < 0.05. *P. clarkii*=*Procambarus clarkii*

## Discussion

The incorporation of PC into the diet of fish and crustaceans has been studied for its potential to enhance pigmentation and immunity. PC, a blue-green pigment found in cyanobacteria like *Spirulina*, is known for its vibrant color and antioxidant, anti-inflammatory, and immune-enhancing properties.

### PC enhances the fat content of diet

The present study indicated that increasing PC concentration in crayfish diets enhances fat content, possibly due to the lipid fraction in the PC extract itself. These findings are consistent with previous studies that identified lipid components within PC extracts, contributing to the overall fat content in supplemented diets [21, 22]. Despite this increase in fat content, the protein levels across all experimental sets remained relatively stable, suggesting that PC supplementation does not significantly impact the protein composition of crayfish. This consistency in protein levels supports the view that the primary function of PC in the diet is related to pigmentation and possibly immune enhancement rather than nutritional content alteration [23].

Variations in moisture content were observed in this study, particularly in the control and 500 mg/kg PC groups, which had higher moisture levels. These fluctuations in moisture may be attributed to differences in pellet preparation or storage conditions, which may affect the hygroscopic properties of the feed [24]. The variability in moisture content underscores the importance of maintaining consistent production and storage protocols to ensure the stability of the dietary formulation over time.

### PC enhances pigmentation and immune system function in aquatic animals

PC acts as a natural pigment that significantly enhances the coloration of fish and crustaceans, which is particularly important in ornamental aquaculture, where the vibrancy of color directly impacts market value. The pigment is deposited on the skin and scales, enhancing hues, particularly in red and blue tones. Studies have shown that PC supplementation increases the a* (redness) and b* (yellowness) values of various species, such as koi carp (*C. carpio* var. *koi*), betta fish (*Betta splendens*), and red swamp crayfish (*P. clarkii*), thereby improving their esthetic appeal [25, 26]. This effect is dose-dependent, with higher PC concentrations typically resulting in more pronounced color changes.

In addition to its role in pigmentation, PC enhances the immune system of fish and crustaceans. The antioxidant properties of PC help mitigate oxidative stress by neutralizing free radicals and reactive oxygen species accumulation in cells caused by environmental stressors in aquaculture, which is a common issue in aquaculture environments. By reducing oxidative stress, PC protects cells from degradation, supports immune system function, and helps maintain overall cellular integrity, which is essential for fish and crustaceans’ health and resilience in intensive farming environments. PC stimulates the production of cytokines and other immune-related molecules, thereby enhancing the ability of organisms to resist diseases [26, 27]. The inclusion of PC in the diet can improve the immune response, which is crucial for maintaining the health, survival, and productivity of aquaculture species.

The dual benefits of PC, such as enhanced pigmentation and improved immunity, make them a valuable ingredient in fish and crustacean diets. The pigment enhances color in ornamental fish and protects against common pathogens [28]. PC has been linked to better survival and faster recovery from stress-induced conditions in shrimp [29].

PC enhances pigmentation in crustaceans through multiple pathways, including its role as a light-harvesting pigment and its interaction with the metabolic pathways responsible for the synthesis and deposition of pigments, particularly carotenoids, in the exoskeleton and epidermal tissues [21]. PC is a powerful antioxidant, and its presence in the diet can reduce oxidative stress in cells. By mitigating oxidative damage, PC helps maintain the integrity of carotenoid pigments, which are crucial for the formation of red, yellow, and orange hues in crustaceans [30]. Carotenoids, typically derived from dietary sources, are processed in crustacean tissues, where they bind to proteins and are deposited in the exoskeleton and other pigmented areas. The antioxidant properties of PC may enhance their stability and deposition by protecting them from degradation.

The metabolic pathways responsible for pigment deposition in crustaceans are closely linked to protein synthesis. PC, being a protein itself, may enhance the overall protein synthesis machinery within the organism, leading to an increase in the expression of pigment-binding proteins, such as crustaceans, which bind carotenoids and facilitate their incorporation into the exoskeleton [31, 32]. By increasing the availability of these binding proteins, PC indirectly enhances pigmentation intensity and stability.

The present results indicate that PC supplementation has a dose-dependent effect on the color enhancement of crayfish, particularly in terms of increasing redness and maintaining brightness over time. The highest concentrations of PC (100 mg/kg and 500 mg/kg) were most effective at enhancing the L* and a* values, suggesting that PC is a viable natural pigment for improving the visual appeal of ornamental crayfish. However, the effects on yellowness were less pronounced and varied depending on the timing and concentration of PC.

### PC is an indirect growth-promoting agent of *P. clarkii*

In this study, the impact on growth observed in crustaceans fed diets supplemented with PC underscores the specificity of its role in aquaculture nutrition. Although PC is well-documented for its ability to enhance pigmentation and potentially bolster immune responses through its antioxidant properties, its function does not extend to growth promotion in crustaceans. This distinction likely arises from the primary biological roles of PC, which include acting as light-harvesting pigments and powerful antioxidants rather than as sources of essential nutrients required for growth [21, 33].

Like many other aquatic organisms, Crustaceans have specific dietary needs tightly linked to growth, including adequate sources of protein, lipids, and other essential nutrients. Despite its benefits, PC may not contribute to the fulfillment of these growth-related nutritional requirements. The absence of growth enhancement in the present study is consistent with findings from the previous study [34], suggesting that the nutritional composition of PC does not provide the necessary elements to stimulate growth in crustaceans.

The metabolic pathways that crustaceans use for growth may not be significantly influenced by the bioactive compounds in PC, which may explain the consistent lack of growth responses across studies and species. A precise nutritional formulation for crustacean growth may require other supplements or synergistic interactions with PC, which were absent in this study. Therefore, although PC remains a valuable dietary supplement for improving coloration and possibly enhancing immunity, it should not be relied upon as a growth-promoting agent in crustacean aquaculture.

The present results suggest that the addition of PC to *P. clarkii* crayfish diets significantly enhances or hinders growth compared with a standard diet. PC can be used as a color enhancer without negatively impacting crayfish growth rate. However, additional studies are needed to explore other potential benefits or limitations of aquaculture diets, such as the effects of PC on overall health, reproduction, and stress resistance. Further studies are needed to explore the implications of these physical changes on crayfish health and growth.

## Conclusion

This study demonstrated that PC supplementation in crayfish diets significantly enhances color intensity, particularly brightness, redness, and yellowness. The optimal concentrations for each color parameter vary, with 100 mg/kg PC being most effective for brightness, 500 mg/kg PC being most effective for red intensity, and 50 mg/kg PC being most effective for yellow intensity. PC positively impacted *P. clarkii* crayfish weight gain, with significant effects observed in the final weeks, particularly at higher concentrations. These findings highlight the potential of PC as a natural pigment enhancer in aquaculture, offering a means to improve the visual appeal of ornamental crayfish. These results provide valuable insights into the influence of PC supplementation on the nutritional profile of crayfish diets, which may have implications for growth, survival, and health. Further studies are needed to explore the long-term effects of PC on crayfish pigmentation and to investigate whether similar effects are observed in other crustacean species. The potential mechanisms underlying the differential accumulation of PC in epidermal tissues should also be examined to optimize its use in aquaculture.

## Authors’ Contributions

RH: Conceptualized and designed the study and drafted the manuscript. PY: Sample collection and microbiological culturing. PY and RH: Statistical analyses and revised the manuscript. All authors have read and approved the final manuscript.
